# Regional variations in inpatient satisfaction: a nationwide analysis of ministry of health hospitals in Saudi Arabia

**DOI:** 10.3389/fpubh.2026.1882479

**Published:** 2026-09-04

**Authors:** Eid Khulaif Daeab, Mansour A. Almanaa, Yazeed I. Alashban, Asma S. Alrushud, Omar A. Alsadon, Essam S. Almutleb, Ali Alsaqr, Dara Aldisi, Mahmoud M. A. Abulmeaty, Waleed Alqarni, Mourad A. M. Aboul-Soud

**Affiliations:** 1 Department of Clinical Laboratory Sciences, College of Applied Medical Sciences, King Saud University, Riyadh, Saudi Arabia; 2 Molecular and Cell Biology Laboratory, Prince Naif bin Abdul Aziz Health Research Center (PNHRC), College of Dentistry, King Saud University Medical City, King Saud University, Riyadh, Saudi Arabia; 3 Department of Radiological Sciences, College of Applied Medical Sciences, King Saud University, Riyadh, Saudi Arabia; 4 Department of Health Rehabilitation Sciences, College of Applied Medical Sciences, King Saud University, Riyadh, Saudi Arabia; 5 Dental Health Department, College of Applied Medical Sciences, King Saud University, Riyadh, Saudi Arabia; 6 Optometry Department, College of Applied Medical Sciences, King Saud University, Riyadh, Saudi Arabia; 7 Department of Community Health Sciences, College of Applied Medical Sciences, King Saud University, Riyadh, Saudi Arabia; 8 Patient Experience Center, Ministry of Health, Riyadh, Saudi Arabia; 9 Center of Excellence in Biotechnology Research (CEBR), College of Applied Medical Sciences, King Saud University, Riyadh, Saudi Arabia

**Keywords:** health clusters, healthcare quality, inpatient services, Kruskal–Wallis test, ministry of health Saudi Arabia, non-parametric analysis, patient satisfaction, regional disparities

## Abstract

**Introduction:**

Patient satisfaction is a critical indicator of healthcare quality and a key driver for improvement in hospital services. This study aimed to assess the levels and regional variations in inpatient satisfaction across all 20 Ministry of Health (MOH) regional health clusters in Saudi Arabia.

**Methods:**

A nationwide retrospective cross-sectional analysis was conducted using anonymized survey data from 90,867 inpatient respondents collected through the MOH Patient Experience Measurement Program. The dataset captured patient experiences across multiple domains, including overall care, staff hygiene, physician communication, waiting times, and pain management. As data were ordinal (five-point Likert scale), non-parametric Kruskal–Wallis *H*-tests were employed to compare median satisfaction scores across regions, supplemented by *post-hoc* Dunn's tests with Bonferroni adjustment. Effect sizes (epsilon-squared, ε^2^ = H/(n−1)) were calculated for all nine domains, and a Holm–Bonferroni correction was applied across the family of omnibus tests. To identify the domains most strongly associated with the global rating of care, and to partition variation between hospitals and clusters, a proportional-odds ordinal regression and a three-level mixed-effects logistic model with respondents nested within hospitals within clusters were additionally fitted.

**Results:**

Differences between clusters were statistically significant in every domain (all Holm-adjusted *p* < 0.001) but negligible in magnitude (ε^2^ = 0.002–0.020, with ε^2^ < 0.01 in eight of the nine domains). In the adjusted models the strongest correlates of the global rating were non-physician process domains—courtesy at blood draw (proportional-odds ratio 1.51, 95% CI 1.47–1.55) and staff hand hygiene (1.49, 1.45–1.53)—rather than physician communication (1.20–1.22). Between-hospital variance in a top-box global rating (variance-partition coefficient 4.4%) was more than four times the between-cluster variance (1.1%), and the cluster component fell to approximately zero once hospital was included in the model. The Jazan Health Cluster recorded the highest observed ratings in overall care, hand hygiene, and physician communication, whereas the Al-Jouf, Al-Ahsa, and Al-Madinah clusters recorded the lowest. After risk standardization for age band, sex, and survey year, 11 of the 20 clusters were statistically distinguishable from the national mean. Despite regional variations, aggregate data revealed high overall satisfaction, with most patients rating their experience as “Good” or “Very Good”—for example, 72.81% rated staff hand hygiene as “Very Good,” and 74.86% rated physician concern for questions as “Very Good.” While significant inter-regional differences exist, overall patient satisfaction with MOH inpatient services in Saudi Arabia is high.

**Discussion:**

Because most of the modeled variation lies between hospitals rather than between clusters, quality-improvement effort is better targeted at facility level than at cluster level; clusters are accordingly described here as showing higher or lower observed ratings rather than as high- or under-performing. National healthcare policies should focus on standardizing protocols and sharing best practices to ensure equitable, high-quality patient care across all regions.

## 1 Introduction

Patient satisfaction is a cornerstone of healthcare quality assessment, directly influencing clinical outcomes, patient adherence, and hospital reputation (1). In Saudi Arabia, the Ministry of Health (MOH) oversees a vast network of hospitals across 20 geographically and administratively distinct health clusters. Evaluating inpatient satisfaction at a national level is essential for benchmarking performance, guiding resource allocation, and implementing evidence-based quality improvement programs (2). Although patient experience surveys are routinely conducted, a comprehensive, comparative analysis of regional variations across multiple service domains is lacking. This study addresses this gap by providing a detailed examination of inpatient satisfaction levels and their regional disparities within Saudi Arabia's Ministry of Health hospitals (3). Prior research has explored patient satisfaction in Saudi Arabia, often highlighting demographic influences such as gender, education, and age on satisfaction levels with physician services (4). For instance, studies have shown that patient socioeconomic factors, including income and occupation, significantly influence inpatient satisfaction with service quality in Saudi Arabian hospitals (5). However, a nationwide analysis specifically focusing on regional variations in patient satisfaction within the Ministry of Health's extensive hospital network has not been comprehensively conducted to date (6). This study aims to fill this void by analyzing patient satisfaction data from all Ministry of Health hospitals, providing a robust, nationally representative assessment of healthcare quality across various regions (7).

Saudi Arabia's healthcare system is undergoing a profound transformation under Vision 2030, with the Health Sector Transformation Program restructuring service delivery into integrated regional clusters intended to decentralize governance, improve population health management, and align provider incentives with patient-reported outcomes (8). Within this framework, patient experience has been formally adopted as a strategic performance indicator, and standardized inpatient satisfaction surveys are administered routinely across all MOH facilities by the Patient Experience Measurement Program. Conceptually, patient satisfaction is best understood as a multidimensional construct shaped by the convergence of structural inputs (staffing, infrastructure, supplies), process attributes (communication, responsiveness, hygiene, pain management), and patient-level expectations and cultural norms—a framing consistent with Donabedian's structure–process–outcome model and with international evidence linking communication and responsiveness to global ratings of care (9, 10). Geographic variation in any of these inputs—particularly between urban tertiary centers and remote border regions—can therefore translate into measurable disparities in patient-reported experience, even within a single national health system (11).

For clarity, MOH health clusters in Saudi Arabia are integrated service-delivery networks established under the Health Sector Transformation Program. Each cluster aggregates hospitals, primary healthcare centers, and specialty facilities within a defined geographic catchment under unified governance, with a mandate to deliver the full continuum of primary, secondary, and tertiary care to a target population. The 20 clusters span the country and are administratively distinct, ranging from large multi-cluster regions such as Riyadh (three clusters) and Jeddah (two clusters) to smaller single-cluster regions such as Hafr Al-Baten and Northern Borders. This cluster-based organization is the unit of comparison in the present analysis.

A terminological clarification is warranted. Patient satisfaction denotes an evaluative judgment conditioned by prior expectation and cultural norm, whereas patient experience denotes whether specific care processes were reported to have occurred. The instrument analyzed here comprises predominantly experience items (observed hand hygiene, waiting time, explanations received, physician communication) together with a single global evaluative rating. We therefore use “inpatient experience” for the domain items and reserve “satisfaction” for the global rating, and do not treat the two as interchangeable. For the same reason “health cluster” (or “cluster”) is used throughout for the unit of analysis: Riyadh and Jeddah each comprise more than one cluster, so “region” would imply an administrative geography that does not map one-to-one onto service-delivery clusters.

A central enabler of cluster-level performance monitoring is the MOH National Health Command Center (NHCC) in Riyadh, which functions as a nationwide hub for aggregating, visualizing, and analyzing healthcare data using a combination of artificial-intelligence pipelines and domain expertise. Originally scaled up during the COVID-19 response to provide capacity and demand analytics, the NHCC now sustains routine performance monitoring across multiple operational domains—including hospital services, patient transfers, supply-chain management, and patient experience—through interlinked dashboards and a structured ticketing workflow that documents corrective actions and supports interdepartmental accountability. By integrating patient-experience feeds with operational and clinical data streams, the NHCC creates the analytic substrate on which nationwide comparisons such as the present study are made feasible (11).

This comprehensive approach allows for the identification of best practices and areas requiring targeted intervention to enhance the overall patient experience and ensure equitable, high-quality care nationwide (11). Furthermore, understanding the interplay between health status and responsiveness within the healthcare system is crucial, as patients with multiple chronic conditions often exhibit lower satisfaction with various dimensions of outpatient care (12). Comparable nationwide benchmarking studies in other large health systems (e.g., HCAHPS in the United States and the National Inpatient Survey in the United Kingdom) have consistently demonstrated that aggregate national figures mask substantial sub-national heterogeneity, and that exposing such heterogeneity is itself a prerequisite for closing equity gaps in care quality (13, 14). This study aims to provide a detailed, nationwide evaluation of inpatient satisfaction across Saudi MOH hospitals. The specific objectives are to (i) identify, using multivariable ordinal and multilevel models, the domains of care most strongly associated with the global rating of inpatient care; (ii) compare satisfaction levels across the 20 health clusters to uncover regional strengths and weaknesses; and (iii) provide data-driven recommendations for standardizing and elevating the quality of inpatient care nationwide.

## 2 Materials and methods

### 2.1 Study design and setting

A retrospective, nationwide cross-sectional analysis was performed on anonymized patient satisfaction survey data collected from inpatient services across all 20 MOH regional health clusters in Saudi Arabia. The study was applied to inpatients at public hospitals between January 2022 and December 2024. The 20 clusters together cover every administrative region of the Kingdom and operate under a single federal Ministry of Health governance framework, with hospitals classified by level of care (general, specialty, and tertiary referral). The study analyzed inpatient experience indicators routinely captured by the centralized MOH Patient Experience Measurement Program. The total sample comprised 90,867 valid responses.

### 2.2 Data source, sampling, and survey administration

Data were drawn from the centralized Patient Experience Measurement Program maintained by the Saudi Ministry of Health, which is operated as part of the National Transformation Program and conducted in partnership with Press Ganey, an independent third-party patient-experience measurement organization, in order to standardize survey administration and minimize data-collection bias. Within this program, a standardized post-discharge questionnaire is administered to inpatients across all participating MOH hospitals using a uniform field-collection protocol. Patients (or, for minors, their legal guardian) are contacted shortly after discharge and invited to complete the survey.

#### 2.2.1 Sampling frame and time window

Survey responses analyzed in this study were collected between January 2022 and December 2024. Because the Patient Experience Measurement Program operates as a continuous national census of recently discharged inpatients—rather than drawing a probability sample within each cluster—a formal *a priori* sample-size calculation was not performed; instead, all eligible responses received during the data window were included, yielding a final analytic sample of 90,867. Cluster-level response volumes are reported in Table 1 and Supplementary Table S1 and are proportional to each cluster's inpatient throughput.

**Table 1:** Demographic profile of survey respondents (*N* = 90,867).

Variable	Group	Frequency (*n*)	Percentage (%)
Age	< 18	27,027	29.74
	18–29	21,555	23.72
	30–44	29,069	31.99
	45–59	9,419	10.37
	≥60	3,797	4.18
Gender	Female	24,963	27.66
	Male	65,288	72.34
Health cluster	(Top 5 by %)		
	Aseer health cluster	9,808	10.79
	Riyadh health cluster 1	9,405	10.35
	Al-madinah health cluster	8,194	9.02
	Riyadh health cluster 2	8,073	8.88
	Makkah health cluster	6,467	7.12
	Other 15 clusters	37,803	41.60

Cluster rows show the five largest contributing clusters; full regional breakdown for all 20 clusters is provided in Supplementary Table S1. Age-band percentages are computed on the full analytic *N* = 90,867; sex percentages are computed on the 90,251 records carrying a recorded sex value, since sex was missing for 616 records. Percentages may not sum to 100.0% due to rounding.

#### 2.2.2 Survey administration mode

The questionnaire was administered to discharged inpatients via telephone interview. Responses were submitted in Arabic or English at the patient's preference and stored under unique anonymized identifiers within the central MOH data warehouse. No personally identifying information was released to the research team.

#### 2.2.3 Survey administration and proxy responses

All clusters operated under the same centrally specified field protocol, delivered by a single measurement partner, with Arabic and English administration available throughout. For respondents aged < 18 years a legal guardian completed the survey on the patient's behalf; such records constitute 29.74% of the sample and were retained in the primary analysis, with age band (which separates guardian-completed from self-completed records) entered as a covariate in every adjusted model. The interval between discharge and contact, and the number of contact attempts made before a record was classified as non-contacted, are not retained in the de-identified extract released to the research team and therefore cannot be reported.

#### 2.2.4 Response rate

The Patient Experience Measurement Program does not retain a cluster-level denominator of eligible discharged inpatients contacted, so a conventional response rate cannot be computed for this dataset. This is treated as a substantive limitation (Section 4.5): without the denominator, differential non-response between clusters cannot be excluded as a partial explanation for the differences reported below.

#### 2.2.5 Inclusion and exclusion criteria

Eligible respondents were inpatients aged ≥ 1 year (or their legal guardian, in the case of minors) who completed an inpatient episode at any MOH facility during the data-collection window and who consented to participate in the post-discharge survey. Records were excluded prior to analysis if they had (i) missing region or cluster code, (ii) implausible age values, (iii) duplicate respondent identifiers, or (iv) fully blank response sets across all surveyed domains (15). Day-case and same-day discharge records were not included in the inpatient measurement program and therefore did not enter the analytic dataset.

### 2.3 Survey instrument and validation

The questionnaire used by the Patient Experience Measurement Program consists of items adapted from internationally validated patient-experience instruments—most notably the Hospital Consumer Assessment of Healthcare Providers and Systems (HCAHPS) family of surveys (13, 15)—translated into Arabic and culturally adapted for the Saudi context by the Patient Experience Center, Ministry of Health. The instrument captures inpatient experience along multiple service domains using a five-point Likert response scale. Within this study we analyzed the nine domains for which complete cluster-level data were available (Table 2). Formal psychometric validation (e.g., factor structure, internal consistency, test–retest reliability) of the Arabic-language MOH version was conducted in line with Press Ganey's system of standardized survey-validation protocols.

**Table 2:** Results of Kruskal–Wallis *H*-tests for key patient satisfaction domains.

Satisfaction domain	*H* (df; item *n*)	*p* (Holm-adjusted)	Effect size, ε^2^	Clusters with higher observed ratings	Clusters with lower observed ratings
Overall care rating	648.42 (19; 80,397)	< 0.001	0.008	Jazan, Ta'if, Northern Borders	Al-Jouf, Al-Ahsa, Al-Madinah
Staff hand hygiene	386.80 (19; 79,736)	< 0.001	0.005	Jazan, Jeddah 2, Riyadh 2	Al-Bahah, Al-Jouf, Al-Madinah
Courtesy during blood collection	310.22 (19; 70,137)	< 0.001	0.004	Jazan, Ha'il, Al-Qaseem	Al-Madinah, Al-Ahsa, Al-Jouf
Explanations about tests	303.34 (19; 82,424)	< 0.001	0.004	Jazan, Ta'if, Jeddah 2	Al-Jouf, Al-Bahah, Al-Madinah
Waiting time for tests	262.08 (19; 82,226)	< 0.001	0.003	Northern Borders, Jazan, Ta'if	Al-Ahsa, Al-Bahah, Al-Jouf
Physician's concern for questions	213.02 (19; 83,563)	< 0.001	0.003	Jazan, Ta'if, Riyadh 2	Al-Jouf, Al-Madinah, Aseer
Physician keeping patient informed	211.33 (19; 83,406)	< 0.001	0.003	Jazan, Ta'if, Eastern	Al-Jouf, Al-Madinah, Aseer
Time physician spent with patient	166.75 (19; 83,503)	< 0.001	0.002	Jazan, Ta'if, Eastern	Al-Jouf, Al-Madinah, Al-Bahah
Labor pain management	91.42 (13; 4,577)	< 0.001	0.020	Riyadh 2, Al-Qaseem, Al-Jouf	Makkah, Al-Ahsa, Jeddah 2

All nine omnibus tests remained significant at *p* < 0.001 after Holm–Bonferroni adjustment across the family of nine tests; exact p-values ranged from 2.98 × 10^−125^ (overall care) to 7.49 × 10^−14^ (labor pain management). Degrees of freedom are *k*−1; *k* = 20 for all domains except labor pain management, which was answered in only 14 clusters. Effect sizes are ε^2^ = *H*/(*n*−1) computed on the item-level valid response count shown in the second column; the η^2^*H* analog gave equivalent values (0.0018–0.0172) and the same ordering. All effect sizes were negligible (ε^2^ < 0.01) except labor pain management (ε^2^ = 0.020, small). Bonferroni-adjusted Dunn's *post-hoc* tests were used for pairwise comparisons within each domain (Supplementary Table S2). Because the comparison is not risk-adjusted, the final two columns report the three clusters with the highest and lowest mean ranks and must not be read as identifying high- or under-performing clusters; risk-standardized estimates are given in Table 3. The cluster attributions in this table differ from those in the originally submitted version, which were carried over from a preliminary analysis.

The validation dossier for the Arabic-language MOH version is held proprietarily by the measurement partner and was not available for independent inspection. We are consequently unable to report the translation and back-translation procedure, item-level factor loadings, Cronbach's α, test–retest coefficients, or evidence of equivalence between administration modes, and we did not test measurement invariance across language, cluster, sex, or age group. Absent demonstrated invariance, part of any observed between-cluster difference may reflect differential response style rather than differential experience; this is stated as a limitation in Section 4.5.

### 2.4 Variables and measures

The analysis focused on nine key service domains derived from survey items: overall rating of hospital care (ADAA); Staff hand hygiene before examination (I92); Courtesy during blood collection (I269); Waiting time for tests/treatments (T1); Explanations about tests/treatments (T3); How well the physician kept the patient informed (P3); Physician's concern for questions/worries (P2); Time physician spent with patient (P1); and Pain management during labor (L48). Responses were recorded on a five-point Likert scale (1 = Very Bad, 2 = Bad, 3 = Fair, 4 = Good, 5 = Very Good) and treated as ordinal variables. Demographic variables (age, gender, region) were also analyzed. The labor pain management item (L48) was applicable only to female respondents who underwent labor during the index admission, which explains its substantially smaller eligible sub-sample (*N* = 4,577).

### 2.5 Statistical analysis

Given the ordinal nature of the data, distributions were treated as non-normal. Descriptive statistics (frequencies, percentages) summarize demographic and response profiles. The primary inferential analysis used the non-parametric Kruskal–Wallis H-test to determine whether median satisfaction scores differed significantly across the 20 health clusters for each service domain. Where the omnibus test was significant (*p* < 0.05), *post-hoc* pairwise comparisons were conducted using Dunn's test with Bonferroni adjustment to identify specific between-cluster differences. Because nine omnibus tests were conducted, one per domain, a Holm–Bonferroni correction was additionally applied across that family of tests; all nine remained significant at *p* < 0.001 after adjustment. The Bonferroni adjustment applied to Dunn's pairwise comparisons operates within domains and is reported separately from this family-wise adjustment across domains. Effect sizes were calculated for every domain as epsilon-squared, ε^2^ = *H*/(*n*−1), where n is the item-level valid response count rather than the full analytic *N*; the eta-squared analog, η^2^*H* = (*H*–*k* + 1)/(*n*–*k*), was computed alongside it and gave equivalent conclusions. Values of ε^2^ ≈ 0.01 were treated as small, ≈0.06 medium, and ≥0.14 large in accordance with conventional benchmarks (16); values below 0.01 are reported as negligible. The sex distribution was summarized with a 95% confidence interval for the male proportion. A one-proportion *Z*-test against an equal-sex null was also computed, using as its denominator the 90,251 records carrying a recorded sex value rather than the full analytic *N*, because sex was missing for 616 records; the test is reported for completeness only, since a 50:50 null has no substantive interpretation for an administrative inpatient population. Given the very large sample size (*N* = 90,867), *p*-values were interpreted cautiously and always in conjunction with effect sizes, since trivial differences can reach statistical significance in samples of this magnitude (17). Analyses were conducted in Python 3 within a Jupyter Notebook environment using pandas, NumPy, SciPy (scipy.stats), scikit-posthocs (Dunn's test), and Matplotlib/Seaborn for visualization. Two-tailed tests with α = 0.05 were used throughout.

#### 2.5.1 Multivariable analysis of the global rating

To address objective (i), a proportional-odds ordinal logistic regression was fitted with the overall rating of care (ADAA) as the ordinal outcome and the seven general service domains (I92, I269, T1, T3, P3, P2, P1) as predictors, each entered as a one-point increment on its five-point scale, together with age band, sex, and survey year. The obstetric item L48 was excluded from this model because it applies to only a small sub-population. Coefficients are reported as proportional-odds ratios with 95% confidence intervals. The proportional-odds assumption was examined by refitting the model as a series of binary logistic regressions at each of the four cut points and comparing the resulting item odds ratios.

#### 2.5.2 Multilevel variance partition

To separate compositional from cluster-level explanations, mixed-effects logistic models were fitted to a binary top-box outcome (“Very Good” vs. all other responses on ADAA), with random intercepts for health cluster, for hospital, and for both simultaneously in a nested three-level specification of respondents within hospitals within clusters. Models were estimated by variational Bayes and variance-partition coefficients computed on the latent logistic scale, taking the level-one variance as π^2^/3. The nested model was then refitted with the seven domain items added, to establish how much of the higher-level variance is attributable to differences in reported process experience.

#### 2.5.3 Risk-standardized cluster estimates

Cluster performance was additionally expressed as a risk-standardized observed-to-expected (O/E) ratio for a top-box global rating, where the expected count was predicted from a logistic model containing age band, sex, and survey year but no cluster or hospital term. Confidence intervals were computed on the log scale, and clusters whose intervals included unity were classified as not distinguishable from the national mean. Unadjusted and risk-standardized rankings are compared in Table 3.

**Table 3:** Survey items and corresponding codes analyzed.

Survey question (Domain)	Code in dataset
Overall rating of care given at hospital	ADAA
Staff hand hygiene before examination	I92
Courtesy of the person who took your blood	I269
Waiting time for tests or treatments	T1
Explanations about what would happen during tests and treatments	T3
How well physician kept you informed	P3
Physician's concern for your questions and worries	P2
Time physician spent with you	P1
How well was your pain managed during labor (including epidural injection)	L48

Codes correspond to dataset variable names used by the MOH Patient Experience Measurement Program. Item L48 was applicable only to female respondents who underwent labor during the index admission. Full regional breakdown is available in the Supplementary Materials.

#### 2.5.4 Sensitivity analyses for ceiling effects and separate treatment of the obstetric item

Because the response distributions were compressed toward the upper end of the scale, cluster comparisons were repeated using two binary re-specifications: a top-box contrast (“Very Good” vs. all other responses) and a negative-experience contrast (“Very Bad,” “Bad,” or “Fair” vs. “Good” or “Very Good”). Item L48 was analyzed separately from the general domains throughout, since it applies only to the obstetric sub-population (*n* = 4,577) and was answered in only 14 of the 20 clusters. Duplicate respondent identifiers were removed before analysis; because identifiers were anonymised prior to release, repeated admissions by the same individual under different anonymised identifiers cannot be excluded, and this residual possibility is acknowledged as a limitation. Modeling was performed in Python 3 using statsmodels.

### 2.6 Ethics statement

The study was conducted in accordance with the principles of the Declaration of Helsinki and was reviewed and approved by the Institutional Review Board of the Ministry of Health, Kingdom of Saudi Arabia (IRB-MOH; National Registration with NCBE-KACST: H-01-R-009; IRB log No. 25–45 M; date of approval: 10 April 2025; category: Expedited). The research involved secondary analysis of fully anonymized, aggregated patient-experience survey data routinely collected by the Saudi Ministry of Health for service-quality monitoring; the IRB therefore granted a waiver of individual informed consent on the basis that no personally identifying information was accessed by the research team and that the dataset posed no greater than minimal risk to participants. Permission to access and analyze the dataset was further granted by the Patient Experience Center, Ministry of Health, Riyadh, Saudi Arabia.

## 3 Results

### 3.1 Demographic profile

The respondent pool (*N* = 90,867) was predominantly male (65,288 of the 90,251 records carrying a recorded sex value, 72.34%; sex was missing for 616 records), with a broad age distribution (Table 1).

The age distribution for inpatients was as follows: under 18 years (29.74%), 18–29 years (23.72%), 30–44 years (31.99%), 45–59 years (10.37%), and 60 years or older (4.18%). The gender distribution was significantly skewed toward male respondents (65,288/90,251 = 72.34%, 95% CI 72.05–72.63%; one-proportion Z = 134.23, *p* < 0.001), reflecting the demographic patterns of the inpatient population surveyed. The substantial proportion of pediatric respondents (< 18) is consistent with parent/guardian-completed surveys for hospitalized children, which is standard practice in this measurement program (15).

### 3.2 Regional variations in key satisfaction domains

The Kruskal–Wallis tests revealed statistically significant differences between clusters for every service domain analyzed (all *p* < 0.001; Table 4), although the accompanying effect sizes were negligible in eight of the nine domains (ε^2^ < 0.01). Between-cluster differences were therefore consistent in direction but small in magnitude, which is what would be expected when an ordinal outcome with a pronounced ceiling is tested in a sample approaching 91,000.

**Table 4:** Aggregate frequency distribution of inpatient satisfaction across the surveyed domains.

Survey question	*N*	Very bad (%)	Bad (%)	Fair (%)	Good (%)	Very good (%)	Combined positive (%)
Overall rating of care given at hospital	80,397	1.80	5.12	7.61	37.33	48.14	85.47
Staff hand hygiene before examination	79,736	2.41	0.99	4.85	18.93	72.81	91.74
Courtesy of the person who took your blood	70,137	2.55	0.90	4.65	22.09	69.81	91.90
Waiting time for tests or treatments	82,226	4.59	1.62	7.41	24.77	61.61	86.38
Explanations about tests and treatments	82,424	3.10	1.22	5.90	23.22	66.57	89.79
How well physician kept you informed	83,406	3.32	1.35	5.08	18.23	72.03	90.26
Physician's concern for your questions and worries	83,563	3.17	1.13	4.76	16.08	74.86	90.94
Time physician spent with you	83,503	3.20	1.37	5.89	19.32	70.23	89.55

Percentages are based on the eligible respondent *N* for each item (item-level missingness accounts for inter-item variation in *N*). “Combined Positive” denotes the sum of Good and Very Good responses. The overall rating of care (ADAA), omitted from this table in the originally submitted version, is now reported in the first row, so that the domain carrying the largest omnibus statistic and serving as the outcome of the adjusted models is accompanied by its own denominator and distribution. It is the least ceilinged item in the instrument and the only one in which “Bad” responses outnumber “Very Bad” responses. The labor pain management item (L48; *N* = 4,577; 7.52% Very Bad, 2.01% Bad, 6.69% Fair, 18.68% Good, 65.11% Very Good; 83.79% combined positive) is reported separately given its restricted eligible population and its availability in only 14 clusters. Row percentages may not sum exactly to 100.0% due to rounding.

#### 3.2.1 Labor pain management

**Figure 1:**
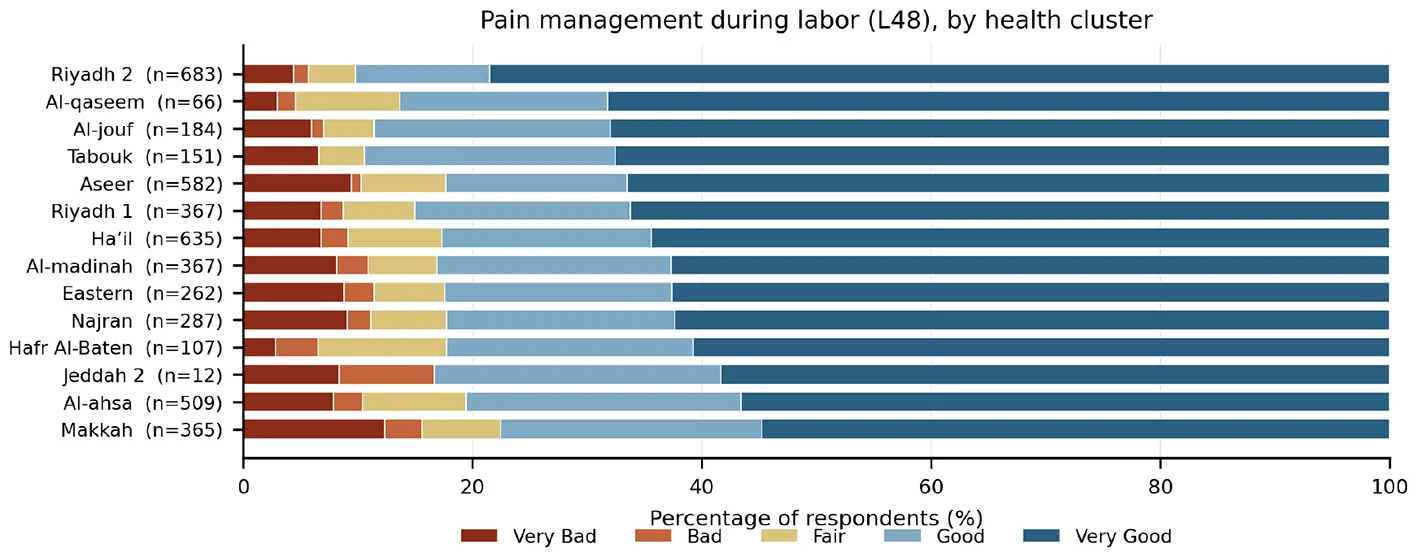
Distribution of responses to the labor pain management item (L48) by health cluster, shown as stacked percentages of the five response categories. Clusters are ordered by the proportion of “Very Good” responses and the eligible n is given beside each cluster. Only 14 of the 20 clusters recorded eligible obstetric respondents. Stacked category bars replace the box plots of the original submission, which conveyed little information because the ratings are ordinal with a pronounced ceiling.

Figure 1 shows the distribution of responses to the labor pain management item by cluster. Ratings differed significantly between clusters [*H*(13) = 91.42, *p* < 0.001], with a small effect size (ε^2^ = 0.020; η^2^*H* = 0.017). This item was answered in only 14 of the 20 clusters, so the omnibus test carries 13 rather than 19 degrees of freedom; the six clusters with no eligible obstetric respondents were Al-Bahah, Jazan, Jeddah 1, Northern Borders, Riyadh 3, and Ta'if. The highest observed ratings were in Riyadh 2, Al-Qaseem, and Al-Jouf and the lowest in Makkah, Al-Ahsa, and Jeddah 2. *Post-hoc* Dunn's tests indicated that Riyadh Health Cluster 2 differed significantly from several other clusters, while most pairwise contrasts were not statistically significant.

#### 3.2.2 Overall care rating

**Figure 2:**
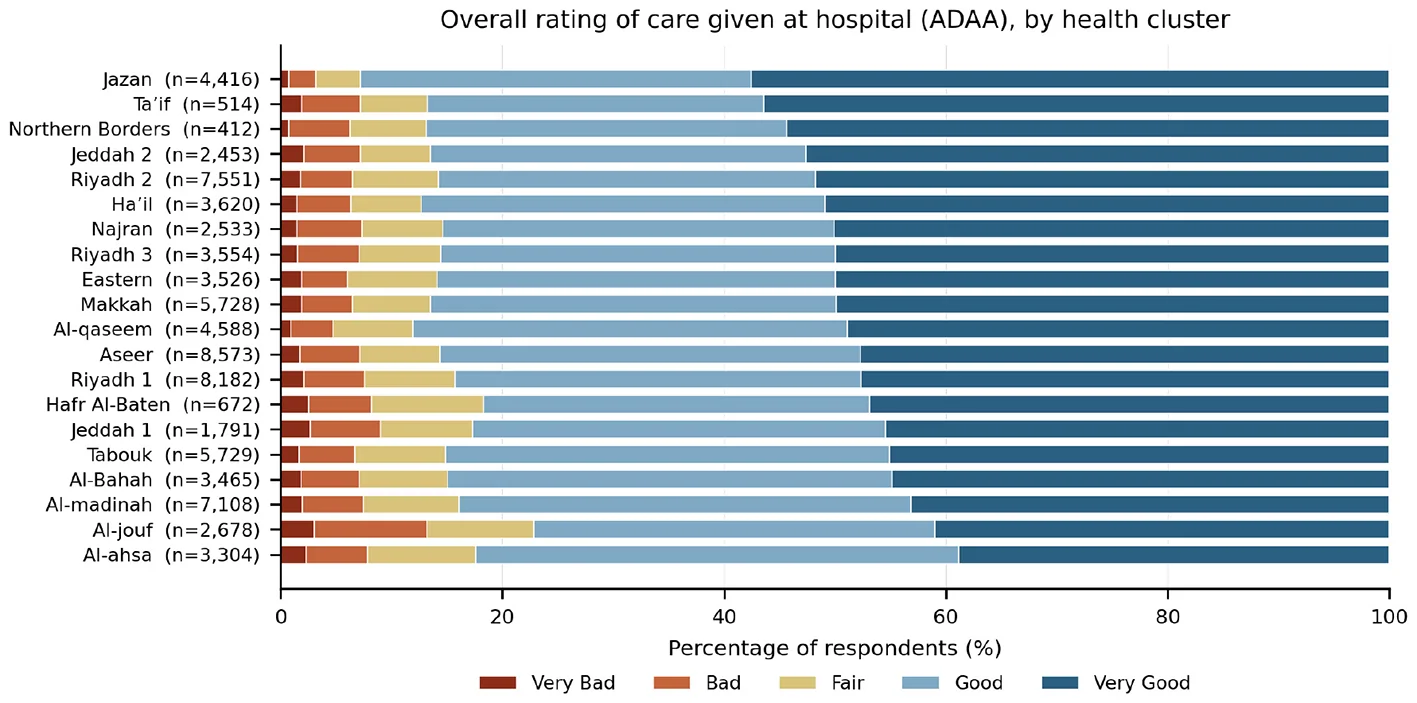
Distribution of responses to the overall rating of care given at hospital (ADAA) by health cluster, shown as stacked percentages of the five response categories, ordered by the proportion of “Very Good” responses.

The largest omnibus statistic was obtained for the overall rating of care [*H*(19) = 648.42, *p* < 0.001; ε^2^ = 0.008, *n* = 80,397]. Ranked by mean rank, the highest observed ratings were in the Jazan, Ta'if, and Northern Borders clusters and the lowest in the Al-Jouf, Al-Ahsa, and Al-Madinah clusters (Figure 2). The proportion of respondents selecting “Very Good” ranged from 38.89% in Al-Ahsa to 57.54% in Jazan, against a national figure of 48.14%. We note that this attribution differs from the cluster labels reported in the originally submitted manuscript, which were carried over from a preliminary analysis; the values above are those obtained from the analytic dataset and supersede them.

#### 3.2.3 Staff hand hygiene

**Figure 3:**
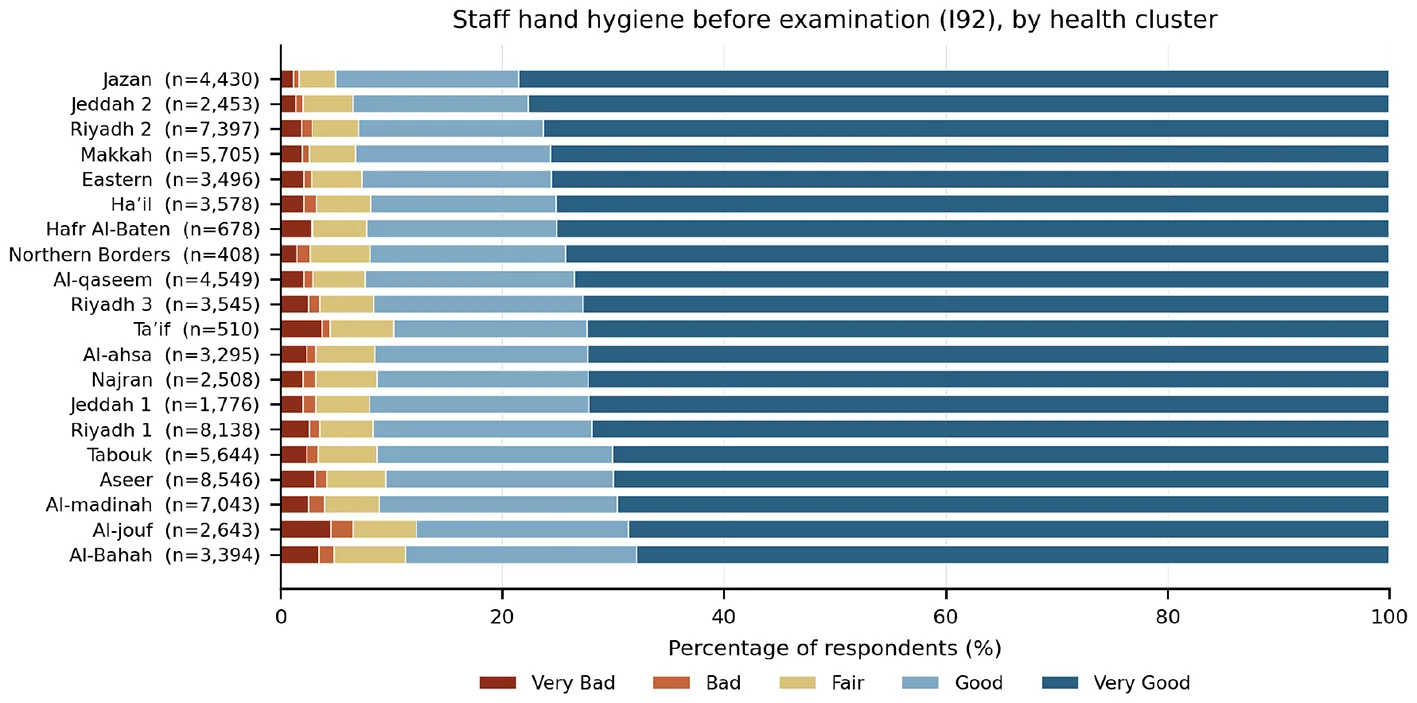
Distribution of responses to the staff hand hygiene item (I92) by health cluster, shown as stacked percentages of the five response categories, ordered by the proportion of “Very Good” responses.

Ratings for staff hand hygiene also varied significantly between clusters [*H*(19) = 386.80, *p* < 0.001; ε^2^ = 0.005], as shown in Figure 3. The highest observed hygiene ratings were in the Jazan, Jeddah 2, and Riyadh 2 clusters and the lowest in Al-Bahah, Al-Jouf, and Al-Madinah.

#### 3.2.4 Physician communication and empathy

**Figure 4:**
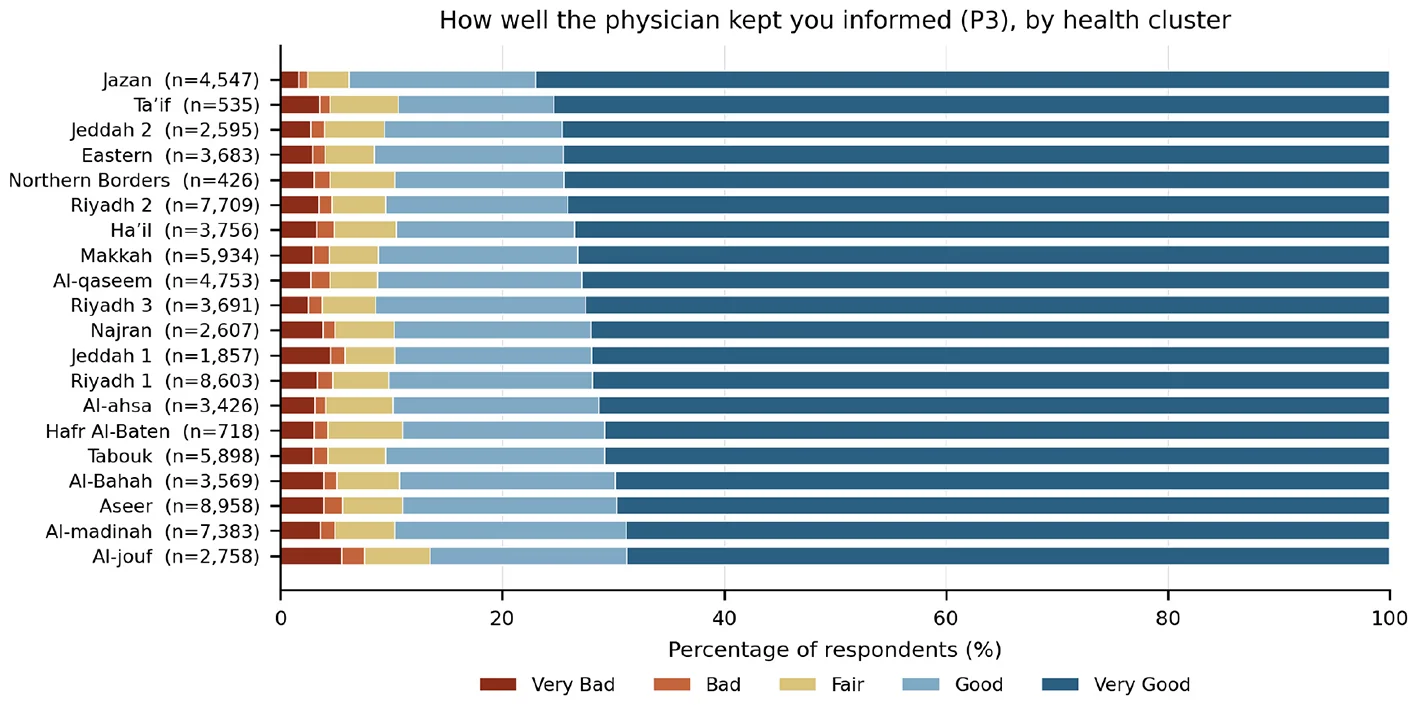
Distribution of responses to the “how well the physician kept you informed” item (P3) by health cluster, shown as stacked percentages of the five response categories, ordered by the proportion of “Very Good” responses.

Significant cluster-level differences were observed for both physician communication items: how well physicians kept patients informed [*H*(19) = 211.33, *p* < 0.001; ε^2^ = 0.003] and physician concern for patient questions [*H*(19) = 213.02, *p* < 0.001; ε^2^ = 0.003]. Figure 4 shows the cluster-level distribution for the “physician kept you informed” item (P3); the corresponding distribution for the “physician concern” item (P2) follows a comparable pattern and is presented in the Supplementary Materials. Jazan and Ta'if recorded the highest observed ratings on both physician items, with the Eastern cluster third on patient-information delivery and Riyadh 2 third on physician concern; Al-Jouf, Al-Madinah, and Aseer recorded the lowest observed ratings on both.

#### 3.2.5 Domains not previously reported

Four domains analyzed in the study had not been tabulated in the originally submitted manuscript and are now reported in full in Table 4. Courtesy during blood collection differed between clusters with the third-largest omnibus statistic of the nine domains [H(19) = 310.22, *p* < 0.001; ε^2^ = 0.004; highest in Jazan, Ha'il, and Al-Qaseem, lowest in Al-Madinah, Al-Ahsa, and Al-Jouf]. Explanations about tests and treatments also differed significantly [H(19) = 303.34, *p* < 0.001; ε^2^ = 0.004; highest in Jazan, Ta'if, and Jeddah 2, lowest in Al-Jouf, Al-Bahah, and Al-Madinah], as did waiting time for tests or treatments [H(19) = 262.08, *p* < 0.001; ε^2^ = 0.003; highest in Northern Borders, Jazan, and Ta'if, lowest in Al-Ahsa, Al-Bahah, and Al-Jouf] and time the physician spent with the patient [H(19) = 166.75, *p* < 0.001; ε^2^ = 0.002; highest in Jazan, Ta'if, and Eastern, lowest in Al-Jouf, Al-Madinah, and Al-Bahah]. Waiting time was the least ceilinged of the service domains, with 61.61% of respondents selecting “Very Good”, and time with the physician yielded the smallest omnibus statistic of any domain measured on the full inpatient sample.

### 3.3 Aggregate satisfaction distribution

Despite regional disparities, the aggregate frequency data depict a consistently positive picture of inpatient experience (Table 5). Across the eight domains for which response volumes exceeded 70,000, the proportion of respondents rating their experience as “Good” or “Very Good” ranged from 85.47% (overall rating of care) to 91.90% (courtesy during blood collection). The global rating was the least ceilinged item in the instrument: only 48.14% of respondents selected “Very Good”, against 61.61–74.86% across the individual service domains, and it was the only item for which “Bad” responses (5.12%) outnumbered “Very Bad” responses (1.80%).

**Table 5:** Risk-standardized cluster estimates for a top-box overall rating of care.

Health cluster	*n*	Observed top-box (%)	Risk-standardized O/E (95% CI)	Unadjusted rank	Standardized rank	Distinguishable from national mean
Ta'if	511	56.36	1.20 (1.07–1.35)	2	1	Yes
Jazan	4,391	57.57	1.20 (1.15–1.25)	1	2	Yes
Northern Borders	411	54.50	1.16 (1.02–1.33)	3	3	Yes
Jeddah 2	2,437	52.57	1.09 (1.03–1.15)	4	4	Yes
Riyadh 2	7,550	51.78	1.06 (1.03–1.09)	5	5	Yes
Ha'il	3,615	50.90	1.06 (1.01–1.11)	6	6	Yes
Najran	2,533	50.10	1.05 (0.99–1.10)	8	7	No
Riyadh 3	3,551	49.99	1.04 (0.99–1.09)	9	8	No
Eastern	3,461	50.19	1.03 (0.98–1.08)	7	9	No
Makkah	5,685	49.87	1.02 (0.99–1.06)	10	10	No
Aseer	8,423	47.66	1.01 (0.98–1.04)	12	11	No
Riyadh 1	8,146	47.63	0.99 (0.96–1.02)	13	12	No
Al-Qaseem	4,581	48.92	0.98 (0.94–1.03)	11	13	No
Jeddah 1	1,736	45.85	0.97 (0.91–1.04)	15	14	No
Al-Bahah	3,458	44.88	0.95 (0.90–0.99)	17	15	Yes
Tabouk	5,709	45.07	0.94 (0.91–0.98)	16	16	Yes
Hafr Al-Baten	656	46.49	0.92 (0.82–1.03)	14	17	No
Al-Madinah	7,069	43.03	0.89 (0.86–0.92)	18	18	Yes
Al-Jouf	2,675	40.97	0.86 (0.81–0.91)	19	19	Yes
Al-Ahsa	3,279	39.04	0.83 (0.79–0.88)	20	20	Yes

Observed top-box is the percentage of respondents selecting “Very Good” on the overall rating of care. The risk-standardized observed/expected (O/E) ratio is the ratio of observed to model-predicted top-box responses, the expected count being predicted from age band, sex, and survey year with no cluster or hospital term; confidence intervals are computed on the log scale. Clusters whose interval includes 1.00 are classified as not distinguishable from the national mean. n is the number of respondents with both a valid overall rating and a recorded sex value (79,877 in total). Only the patient characteristics present in the released extract could be adjusted for, so these estimates are partially rather than fully case-mix adjusted.

### 3.4 Domains associated with the global rating of care, and variance attributable to hospital and cluster

The proportional-odds model of the overall rating of care was fitted to the 64,750 respondents with complete data on all seven general service domains, drawn from 219 hospitals across the 20 clusters. Each one-point increase on an individual domain item was associated with the following increase in the odds of a higher overall rating: courtesy during blood collection, proportional-odds ratio 1.51 (95% CI 1.47–1.55); staff hand hygiene, 1.49 (1.45–1.53); waiting time for tests or treatments, 1.40 (1.37–1.43); time the physician spent with the patient, 1.34 (1.30–1.38); explanations about tests and treatments, 1.28 (1.24–1.32); how well the physician kept the patient informed, 1.22 (1.18–1.26); and physician concern for questions and worries, 1.20 (1.16–1.24). All seven were significant at *p* < 0.001. The ordering is notable: the two physician-communication items were the weakest of the seven, while the strongest correlate was courtesy at blood draw. Male respondents reported higher overall ratings than female respondents (1.24, 1.19–1.28), as did respondents aged 45–59 relative to the 18–29 reference group (1.22, 1.15–1.29), whereas the guardian-completed records for patients aged under 18 did not differ significantly from the reference group (1.03, 0.99–1.08). Ratings rose modestly across the study period (2024 vs. 2022: 1.12, 1.07–1.16). Refitting the model as binary logistic regressions at each of the four cut points gave item odds ratios ranging from 1.33 to 1.57 for hand hygiene and from 1.16 to 1.31 for explanations about tests, with the remaining five items varying by less than 0.10 across cut points, indicating that the proportional-odds specification is adequate for the ordering reported here.

The mixed-effects logistic models of a top-box overall rating located the higher-level variation predominantly below the cluster tier. With a cluster random intercept only, the between-cluster standard deviation was 0.189 and the variance-partition coefficient 1.07%. With a hospital random intercept only, the between-hospital standard deviation was 0.389 and the variance-partition coefficient 4.40%, more than four times as large. In the nested three-level specification the hospital component was essentially unchanged (standard deviation 0.388; 4.38%) while the cluster component collapsed to approximately zero (standard deviation 0.010; 0.00%). The between-cluster differences detected by the omnibus tests are therefore attributable almost entirely to differences between the individual hospitals that each cluster happens to contain, rather than to any attribute operating at cluster level. Adding the seven domain items to the nested model reduced the between-hospital standard deviation from 0.388 to 0.299 (variance-partition coefficient 2.64%), indicating that roughly 40% of the between-hospital variance in the global rating is accounted for by differences in reported process experience and the remainder is not.

**Figure 5:**
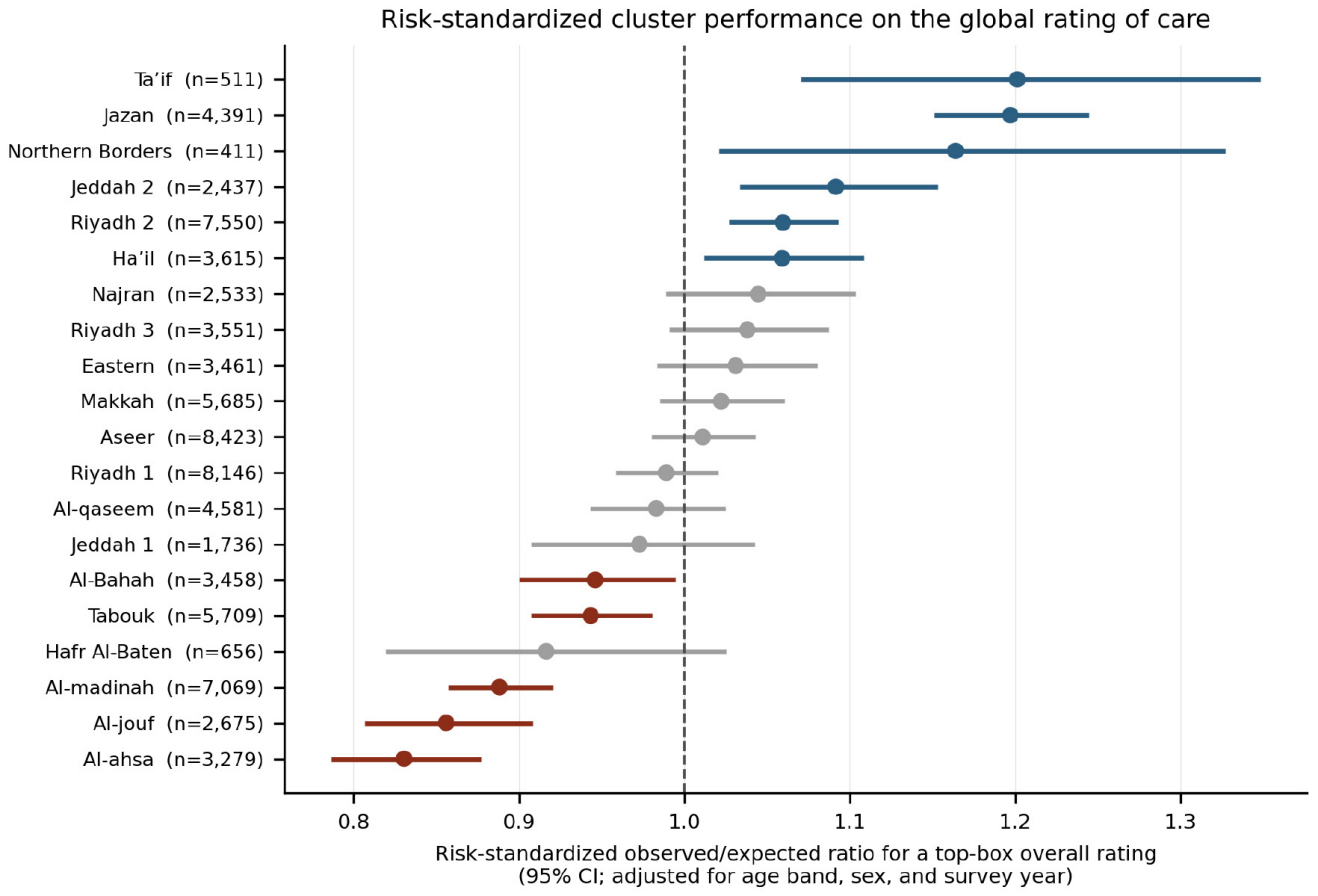
Risk-standardized observed/expected ratios for a top-box overall rating of care, by health cluster, with 95% confidence intervals, adjusted for age band, sex, and survey year. Colored markers denote clusters whose interval excludes 1.00 and which are therefore distinguishable from the national mean; gray markers denote clusters that are not. Numerical values are given in Table 3.

Risk-standardized observed-to-expected ratios for a top-box overall rating are reported in Table 3 and displayed in Figure 5. They ranged from 0.83 in Al-Ahsa (95% CI 0.79–0.88) to 1.20 in Ta'if (1.07–1.35), and 11 of the 20 clusters were statistically distinguishable from the national mean. Standardization for age band, sex, and survey year moved four clusters by two or more rank positions and one by three: Hafr Al-Baten fell from fourteenth on observed ratings to seventeenth after standardization. The unadjusted ordering is therefore not stable even under this limited adjustment, which is the principal reason the final two columns of Table 4 are labeled as observed ratings rather than as performance rankings.

**Figure 6:**
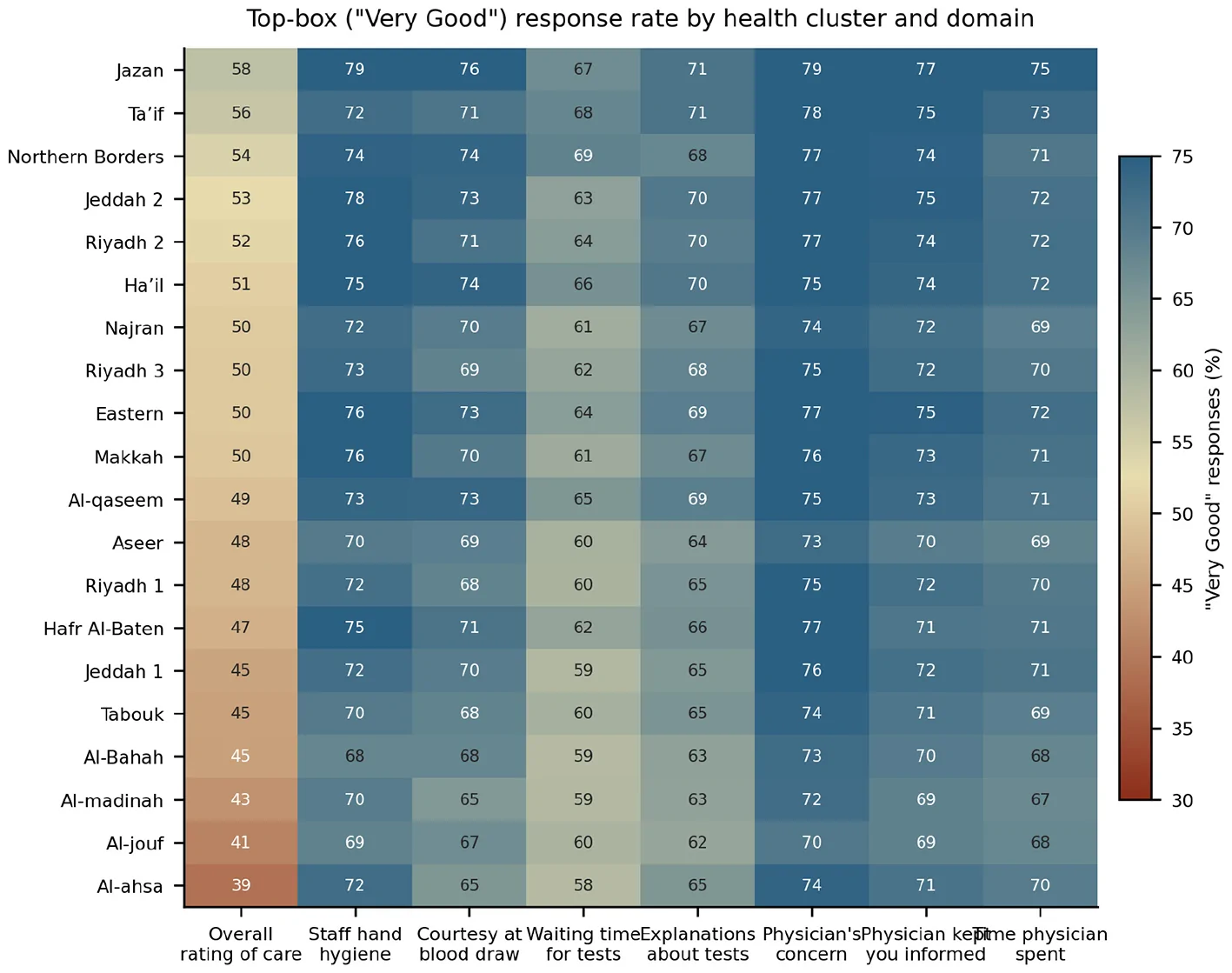
Top-box (“Very Good”) response rate by health cluster and service domain. Cells give the percentage of respondents selecting the highest response category for that cluster and domain, and clusters are ordered by their top-box rate on the overall rating of care. The obstetric item L48 is excluded because it was answered in only 14 clusters.

The top-box and negative-experience decomposition distinguishes two patterns among the clusters with lower observed ratings. Al-Jouf combined a top-box share 7.2 percentage points below the national figure of 48.14% with a negative-response share 8.3 points above the national figure of 14.53%, indicating a substantial tail of poor experiences. Al-Ahsa showed the largest top-box deficit of any cluster, 9.3 points, but an excess of negative responses of only 3.1 points, a profile more consistent with uniformly adequate but unremarkable service than with a concentration of poor experiences. Jazan, conversely, recorded a negative-response share 7.3 points below the national figure. The two profiles call for different interventions, and the distinction is invisible in both the omnibus tests and the median-based comparisons. Figure 6 presents top-box rates for all 20 clusters across the eight general domains.

## 4 Discussion

This nationwide cross-sectional analysis of 90,867 inpatient experiences across all 20 MOH regional health clusters demonstrates two simultaneous and apparently paradoxical findings: aggregate satisfaction with inpatient services in Saudi Arabia is high (~90% “Good” or “Very Good” across most domains), yet statistically significant differences between clusters are present in every service domain examined. The revised analysis reported here adds a third finding that reframes the first two: those differences are negligible in magnitude, and what higher-level variation does exist sits between hospitals rather than between clusters. Patient satisfaction rates in Saudi academic hospitals generally range from 78 to 95.2% across various studies, with technical skill emerging as a primary domain influencing patient satisfaction (3). Our aggregate figures sit in the upper portion of that range and are consistent with the broader Vision 2030 health-sector transformations and ongoing national quality initiatives (8).

### 4.1 Drivers of overall care variation

The most pronounced regional variation was observed in the overall rating of care (*H* = 648.42). Although this was the largest omnibus statistic observed, the corresponding effect size was negligible (ε^2^ = 0.008), and the multilevel analysis locates almost all of the higher-level variation below the cluster tier: the variance-partition coefficient was 4.4% between hospitals but 1.1% between clusters, and the cluster component was indistinguishable from zero once hospital was modeled. Resource allocation, institutional culture, and management effectiveness may well shape global perceptions of care, but the level at which they appear to operate is the individual facility rather than the cluster (18). The overall-care metric is itself driven by upstream domain experiences. In HCAHPS-style models, communication, responsiveness, and cleanliness consistently emerge as the strongest predictors of the global hospital rating (13, 19). Our adjusted model partly diverges from that pattern: the strongest correlates of the global rating were courtesy at blood draw (proportional-odds ratio 1.51, 95% CI 1.47–1.55), staff hand hygiene (1.49, 1.45–1.53), and waiting time for tests or treatments (1.40, 1.37–1.43), whereas the two physician-communication items were the weakest of the seven (physician concern 1.20, 1.16–1.24; physician kept patient informed 1.22, 1.18–1.26). In this setting, therefore, the global rating appears to be shaped more by the courtesy and timeliness of routine service encounters than by the physician consultation itself, a finding with direct implications for where improvement effort is placed. Item odds ratios were broadly stable across the four cut points, supporting the proportional-odds specification, with modest departures for hand hygiene and for explanations about tests that did not alter the ordering of predictors. The consistency with which Jazan and Ta'if appear at the top of multiple domains, and Al-Jouf and Al-Madinah at the bottom, is unlikely to be a chance finding, but its magnitude is small and it may equally reflect differences in case-mix, referral profile, obstetric or pediatric caseload, facility composition, or response style. Two of the clusters previously described as low-performing illustrate the fragility of unadjusted rankings: Northern Borders (*n* = 411 for the global rating) ranked third highest rather than among the lowest, and Hafr Al-Baten (*n* = 656) fell in the middle of the distribution and moved three rank positions on risk standardization (11).

### 4.2 Clinical process and safety

The higher patient-reported hand-hygiene ratings in Jazan, Jeddah 2, and Riyadh 2 are consistent with, though not proof of, more visible infection-control practice. Variation in this fundamental safety metric underscores the need for standardized, mandatory hygiene training and auditing across all regions to minimize infection risk and build universal patient trust (20). Although patient observation is not a substitute for direct compliance auditing, the WHO multimodal hand-hygiene improvement strategy treats patient observation as a complementary indicator that captures the visibility of hygiene practices to patients—an outcome with both safety and trust dimensions (21).

### 4.3 Physician communication and empathy

Jazan and Ta'if recorded the highest observed ratings on both physician-communication items. It is notable, however, that these were the two domains most weakly associated with the global rating in the adjusted model, so communication gaps, while real, are unlikely to be the principal lever on overall satisfaction in this system. Effective communication is a well-documented predictor of overall satisfaction (22). This disparity highlights an opportunity to develop and disseminate a national communication-skills curriculum for clinicians. Moreover, addressing these regional variances through targeted interventions, such as enhanced training programs and resource allocation, could significantly elevate the consistency and quality of patient experiences across all healthcare clusters (23).

### 4.4 Implications for practice and policy

The consistently higher observed ratings of the Jazan Health Cluster across multiple domains identify it as a candidate site for positive-deviance inquiry rather than an established benchmark, since benchmarking presupposes the risk adjustment and facility-level attribution set out below. Conversely, the clusters with the lowest risk-standardized estimates—Al-Ahsa (O/E 0.83, 95% CI 0.79–0.88), Al-Jouf (0.86, 0.81–0.91), and Al-Madinah (0.89, 0.86–0.92)—warrant diagnostic inquiry into the composition of their ratings before intervention is designed. Recommended next steps include:

**(1) Structured quality improvement programs:** implementing tailored action plans in the facilities, rather than the clusters, with the lowest standardized ratings, focusing on courtesy at routine service encounters, hygiene visibility, and waiting times.

**(2) Knowledge transfer:** creating platforms for high-performing clusters to share best practices, protocols, and training modules with others.

**(3) Enhanced monitoring:** deploying real-time satisfaction dashboards at the cluster level to enable prompt managerial response to dips in performance.

**(4) Standardized training:** mandating national training standards for all patient-facing staff in communication skills and clinical safety protocols.

The variance partition reported in Section 3.4 carries a specific policy implication that the originally submitted analysis could not deliver. Because between-hospital variance in the global rating was more than four times the between-cluster variance, and because the cluster component approached zero once hospital was modeled, cluster-level league tables are a poor instrument for improvement in this system: they average over facilities that differ from one another far more than the clusters differ from each other. The unit of accountability for patient experience should therefore be the hospital, with clusters acting as the conduit for support and standard-setting rather than as the object of comparison.

The recommendations above also require disaggregation by level and by domain. At national level, the Ministry of Health is positioned to standardize the measurement instrument and publish its psychometric validation, to report risk-standardized rather than raw scores, to retain the denominator needed for a response rate, to mandate minimum training standards in structured bedside communication, and to define a common hand-hygiene audit specification. At facility level the actionable levers are domain-specific: for waiting time, diagnostic turnaround monitoring, bed-flow management, discharge planning, and test scheduling; for courtesy at blood draw and comparable service encounters, phlebotomy and reception staff training, which the adjusted model identifies as the single strongest correlate of the global rating and which is rarely a target of patient-experience programs; for hand hygiene, direct observation with audit feedback, patient-visible dispenser placement, and safety-culture interventions; and for labor pain management, epidural availability, anesthetic staffing cover, and documented analgesia pathways in the 14 clusters where the item applies.

Finally, the decomposition in Section 3.4 distinguishes two patterns that call for different responses. Al-Jouf combines a low top-box share with a negative-response share 8.3 percentage points above the national figure, indicating a tail of poor experiences to be investigated directly, whereas Al-Ahsa shows the largest top-box deficit (9.3 points below national) with only a modest excess of negative responses, a profile more consistent with uniformly adequate but unremarkable service. Jazan, at the other extreme, has a negative-response share 7.3 points below the national figure. Before any practice is transferred from Jazan or Ta'if, qualitative investigation of why their ratings are higher is required, since staffing, leadership culture, patient mix, infrastructure, survey timing, and local response patterns are not separable in these quantitative data.

### 4.5 Strengths and limitations

The principal strengths of this study are its national scope, large sample size (*N* = 90,867), and inclusion of every MOH regional cluster, which together produce a uniquely representative picture of inpatient experience in the Saudi public hospital system. Several limitations should nonetheless be acknowledged. First, the design is cross-sectional and observational; we cannot infer causal relationships between cluster-level structural features and patient-reported experience. Second, satisfaction surveys are susceptible to non-response bias, social-desirability bias, and ceiling effects—particularly in cultural settings where overtly negative feedback may be discouraged—and these biases may differentially affect clusters and dampen detected differences (24). Third, the sex imbalance (72.34% male) limits the precision with which the experience of female inpatients can be characterized, although this distribution is consistent with the underlying admission profile in MOH inpatient services; sex was in any case adjusted for in the models reported in Section 3.4, where male respondents gave higher global ratings than female respondents. Fourth, with *N* approaching 91,000, even trivial differences achieve statistical significance; we therefore emphasized effect sizes (ε^2^), all of which were negligible to small (ε^2^ = 0.002–0.020), when interpreting the magnitude of between-cluster differences (17). Fifth, the risk standardization applied here uses only the patient characteristics available in the released extract—age band, sex, and survey year. Length of stay, diagnostic category, comorbidity burden, admission route, and language are not present in the dataset, so residual case-mix confounding cannot be excluded and the O/E estimates in Table 3 should be read as partially rather than fully adjusted. Sixth, although the questionnaire is administered under Press Ganey's standardized validation protocols, an independent published psychometric evaluation of the Arabic-language MOH version would strengthen confidence in cross-cluster comparisons. Finally, the data examined here capture inpatient experience and cannot speak to outpatient, emergency, or primary-care satisfaction in the same facilities.

Four further limitations warrant emphasis. First, no response rate can be computed, because the measurement program does not retain the denominator of eligible discharged inpatients contacted; differential non-response between clusters therefore cannot be excluded. Second, the ceiling effect is pronounced in the service domains, where between 86 and 92% of responses fell in the two highest categories, and compressed distributions of this kind limit the capacity of any test to distinguish genuinely higher-performing units from uniformly ceilinged ones; the global rating was materially less ceilinged, which is one reason it was chosen as the outcome for the adjusted models. Third, measurement invariance across language, cluster, sex, and age group was not established, so part of the observed variation may reflect differential response style. Fourth, three clusters contributed fewer than 700 responses to the global rating (Northern Borders, Ta'if, and Hafr Al-Baten), and their estimates carry correspondingly wide intervals; the cluster attributions reported in the originally submitted manuscript were materially affected by this instability, which is the reason the present version reports them from the analytic dataset with confidence intervals attached.

## 5 Conclusion

This nationwide study confirms that while patient satisfaction with inpatient services in Saudi MOH hospitals is generally high, statistically significant but small differences between health clusters are present across all domains of care examined. These findings provide a granular, evidence-based map for quality improvement, and locate the actionable variation principally at the level of the individual hospital rather than the cluster. By investigating the practices of the facilities and clusters with the highest risk-standardized ratings, such as Jazan and Ta'if, and directing diagnostic and improvement effort toward those with the lowest, such as Al-Ahsa, Al-Jouf, and Al-Madinah, the MOH can advance its mission of delivering uniformly excellent, equitable, and patient-centered healthcare across the Kingdom. Future research should employ mixed methods—linking survey data to qualitative interviews with staff and patients, and to structural cluster-level covariates such as bed-to-staff ratios, urbanicity, and accreditation status—to explore the underlying cultural, managerial, and resource-based causes of the observed variations. Longitudinal designs that track these indicators after targeted interventions would also help establish whether benchmark-style knowledge transfer between facilities can demonstrably narrow the differences identified here (25).

## Data Availability

The original contributions presented in the study are included in the article/Supplementary material, further inquiries can be directed to the corresponding author.
