# Supplementary material for: Regional variations in inpatient satisfaction: a nationwide analysis of ministry of health hospitals in Saudi Arabia
Source: Front Public Health. 2026 Sep 4;14:1882479. doi: 10.3389/fpubh.2026.1882479 (PMC13585622; doi:10.3389/fpubh.2026.1882479)
Supplement: Supplementary file 1 [file Data_Sheet_1.zip › Figure S2 — Box plot of Physician Concern_item P2.pdf]

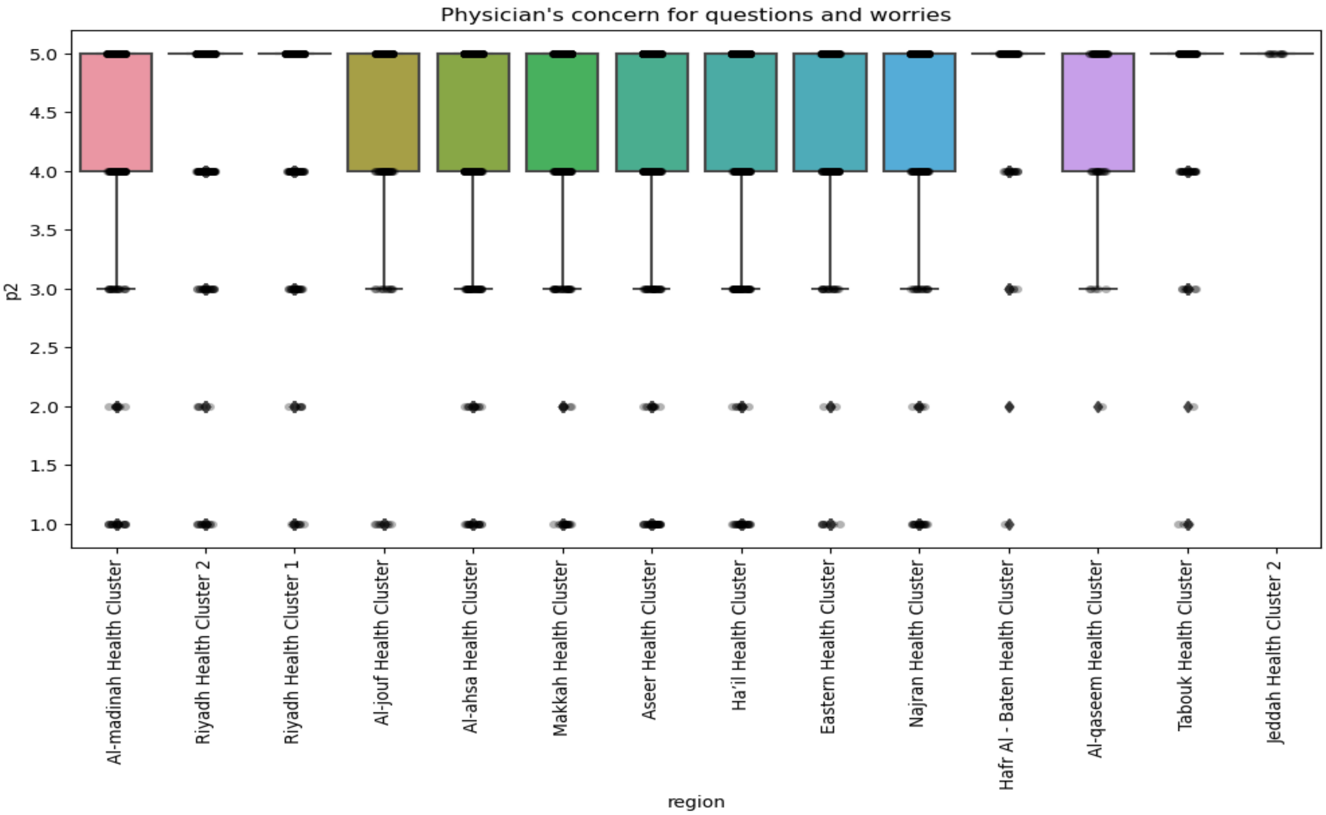

Hypotheses 4:

$H_0$4: The distribution of ratings for staff hand hygiene before examination is the same across all 20 regions.

$H_1$4: The distribution of ratings for staff hand hygiene before examination differs for at least one region.

Calculation:

Kruskal–Wallis H-test

- test statistic = 386.802,
- p-value = 0.0000(~0)

Conclusion:

A Kruskal–Wallis H-test showed a statistically significant difference in patient ratings of staff hand hygiene before examination among the 20 health clusters, $H(19) = 386.802$, $p < .001$. This indicates that patient perceptions of staff hand hygiene vary significantly across regions.
