# Supplementary material for: Regional variations in inpatient satisfaction: a nationwide analysis of ministry of health hospitals in Saudi Arabia
Source: Front Public Health. 2026 Sep 4;14:1882479. doi: 10.3389/fpubh.2026.1882479 (PMC13585622; doi:10.3389/fpubh.2026.1882479)
Supplement: Supplementary file 1 [file Data_Sheet_1.zip › Figure S1 — Cluster-level mean ratings_line plots.pdf]

Figure:

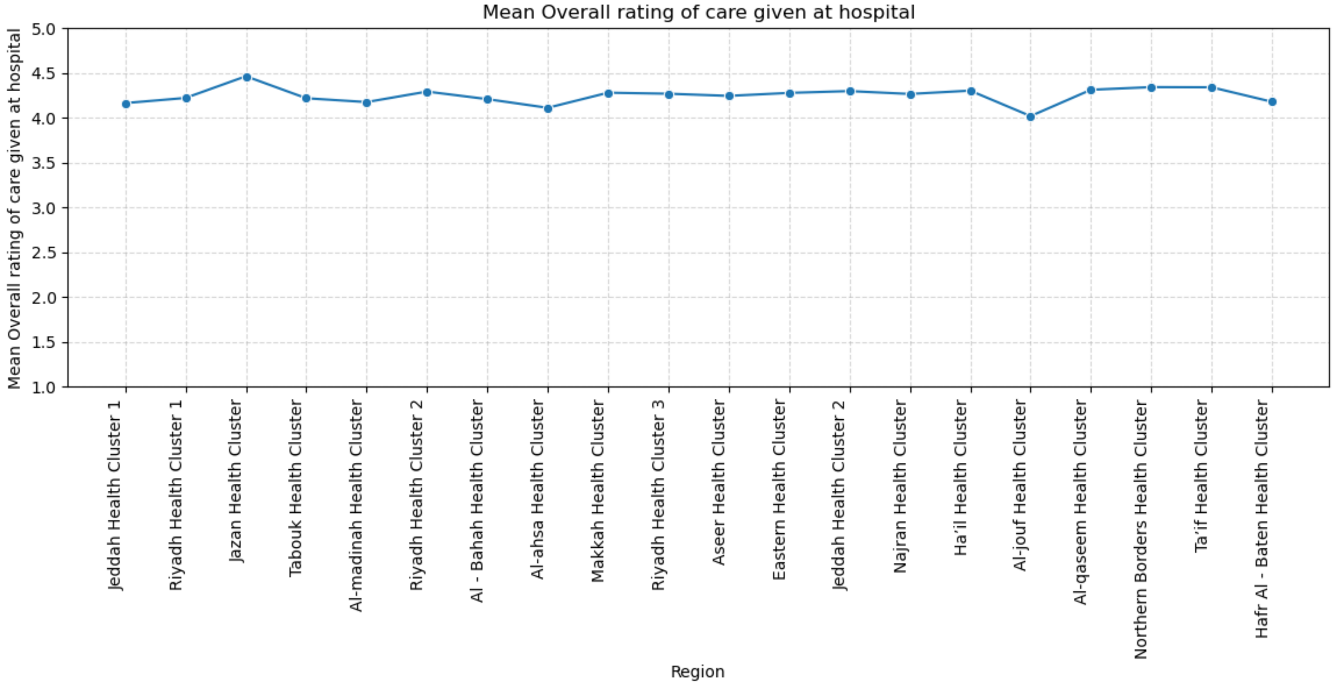
Mean Overall rating of care given at hospital
Mean Overall rating of care given at hospital
5.0
4.5
4.0
3.5
3.0
2.5
2.0
1.5
1.0
Jeddah Health Cluster 1
Riyadh Health Cluster 1
Jazan Health Cluster
Tabouk Health Cluster
Al-madinah Health Cluster
Riyadh Health Cluster 2
Al - Bahah Health Cluster
Al-ahsa Health Cluster
Makkah Health Cluster
Riyadh Health Cluster 3
Aseer Health Cluster
Eastern Health Cluster
Jeddah Health Cluster 2
Najran Health Cluster
Ha'il Health Cluster
Al-jouf Health Cluster
Al-qaseem Health Cluster
Northern Borders Health Cluster
Ta'if Health Cluster
Hafr Al - Baten Health Cluster
Region

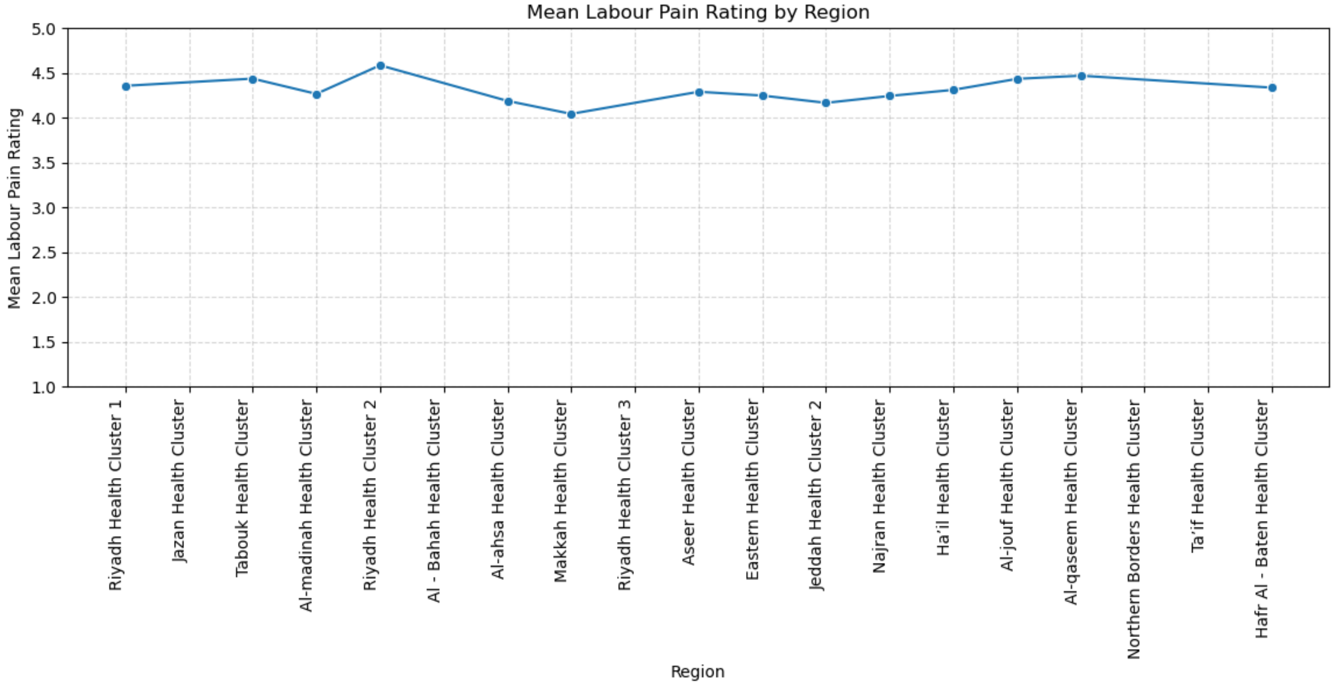
Mean Labour Pain Rating by Region
Mean Labour Pain Rating
5.0
4.5
4.0
3.5
3.0
2.5
2.0
1.5
1.0
Riyadh Health Cluster 1
Jazan Health Cluster
Tabouk Health Cluster
Al-madinah Health Cluster
Riyadh Health Cluster 2
Al - Bahah Health Cluster
Al-ahsa Health Cluster
Makkah Health Cluster
Riyadh Health Cluster 3
Aseer Health Cluster
Eastern Health Cluster
Jeddah Health Cluster 2
Najran Health Cluster
Ha'il Health Cluster
Al-jouf Health Cluster
Al-qaseem Health Cluster
Northern Borders Health Cluster
Ta'if Health Cluster
Hafr Al - Baten Health Cluster
Region

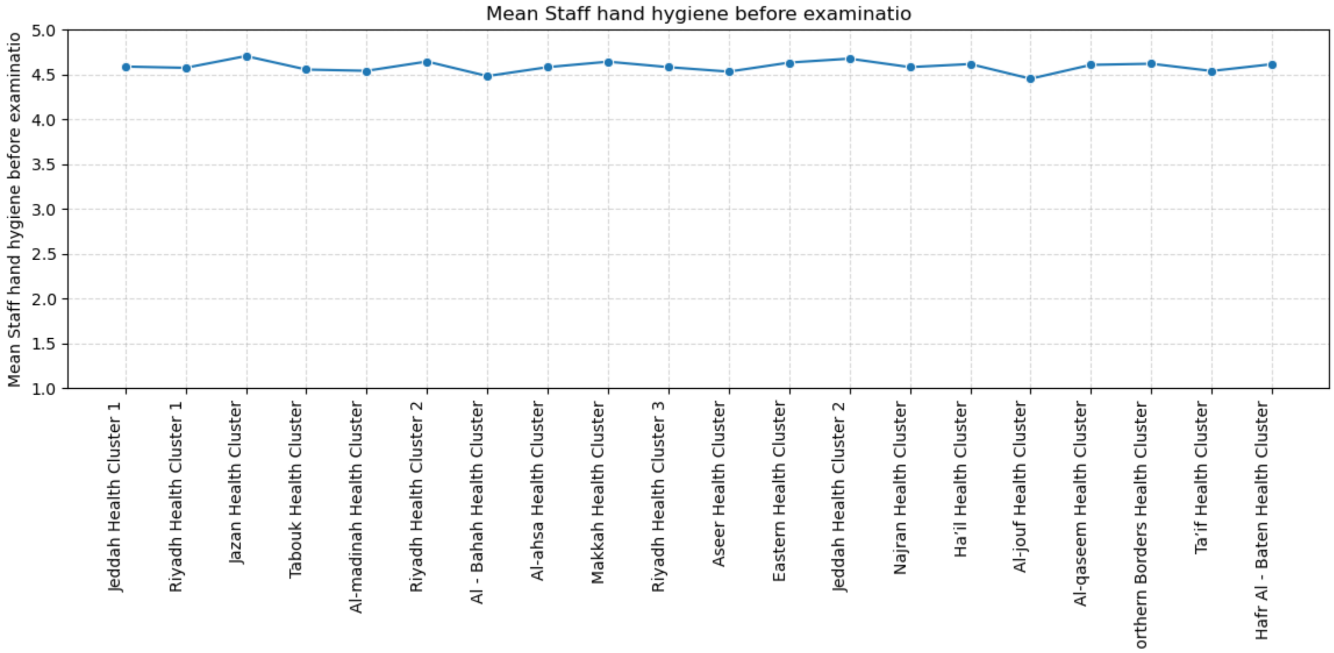

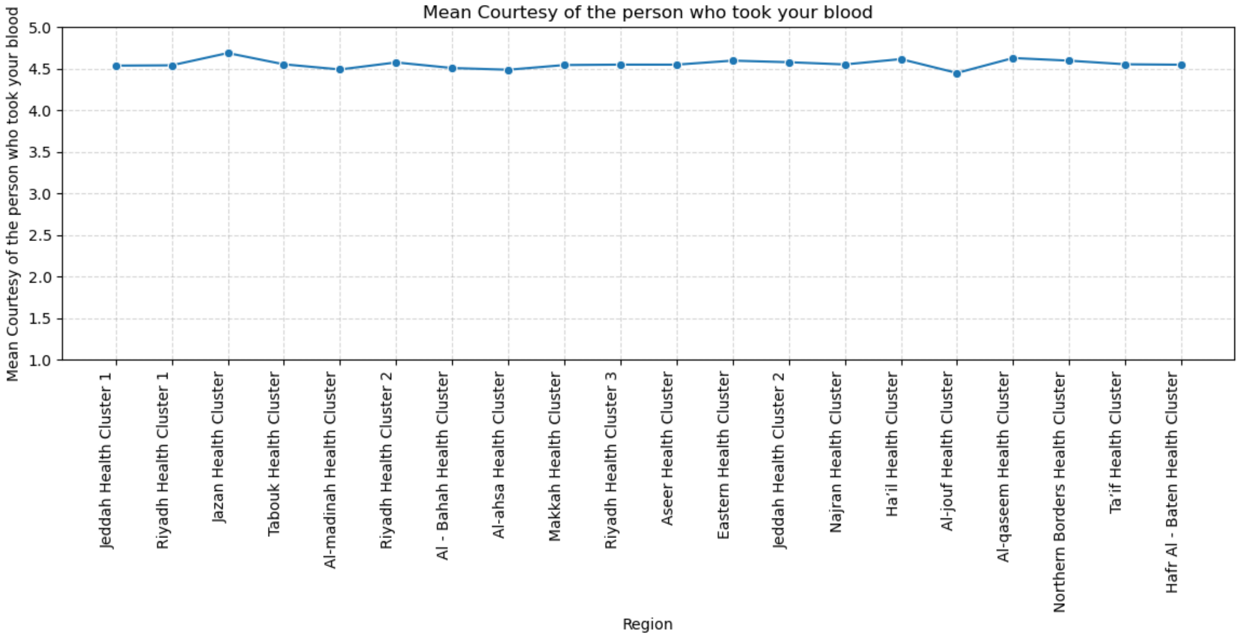

From the above visualization, we can conclude that the **Jazan Health Cluster** has the highest rating among all health clusters, while the **Al-Jouf Health Cluster** has the lowest rating.
